# Validity of the Richmond Agitation-Sedation Scale (RASS) in critically ill children

**DOI:** 10.1186/s40560-016-0189-5

**Published:** 2016-10-26

**Authors:** Abigail Glicksman Kerson, Rebecca DeMaria, Elizabeth Mauer, Christine Joyce, Linda M. Gerber, Bruce M. Greenwald, Gabrielle Silver, Chani Traube

**Affiliations:** 1 Weill Cornell Medical College, New York, NY USA; 2 Department of Healthcare Policy and Research, Weill Cornell Medical College, New York, NY USA; 3 Pediatric Critical Care Medicine, Weill Cornell Medical College, New York, NY USA; 4 Department of Child Psychiatry, Weill Cornell Medical College, New York, NY USA

**Keywords:** Pediatric, Sedation, Critical care, Agitation, Richmond Agitation-Sedation Scale, RASS

## Abstract

**Background:**

The Richmond Agitation-Sedation Scale (RASS) is a single tool that is intuitive, is easy to use, and includes both agitation and sedation. The RASS has never been formally validated for pediatric populations. The objective of this study was to assess inter-rater agreement and criterion validity of the RASS in critically ill children.

**Methods:**

To evaluate validity, the RASS score was compared to both a visual analog scale (VAS) scored by the patient’s nurse, and the University of Michigan Sedation Scale (UMSS), performed by a researcher. The nurse completed the VAS by drawing a single line on a 10-cm scale anchored by “unresponsive” and “combative.” The UMSS was used to validate the sedation portion of the RASS only, as it does not include grades of agitation. For inter-rater agreement, one researcher and the patient’s nurse simultaneously but independently scored the RASS.

**Results:**

One hundred patient encounters were obtained from 50 unique patients, ages 2 months to 21 years. Of these, 27 assessments were on children who were mechanically ventilated and 73 were on children who were spontaneously breathing. In validity testing, the RASS was highly correlated with the nurse’s VAS (Spearman correlation coefficient 0.810, *p* < .0001) and with the UMSS (weighted kappa 0.902, *p* < .0001). Inter-rater agreement between nurse- and researcher-assessed RASS was excellent, with weighted kappa of 0.825 (*p* < .0001).

**Conclusions:**

The RASS is a valid responsiveness tool for use in critically ill children. It allows for accurate assessment of awareness in mechanically ventilated and spontaneously breathing patients, and may improve our ability to titrate sedatives and assess for delirium in pediatrics.

## Background

Critically ill children may experience a range of responsiveness, from coma to extreme agitation, over the course of their intensive care unit (ICU) stay. Most pediatric sedation tools were designed for use in mechanically ventilated patients on pharmacologic sedation and are not easily adaptable to all children in the ICU, regardless of level of respiratory support [1–3]. Few pediatric tools span the continuum of responsiveness, to include agitation as well as sedation [4]. With increasing recognition of the prevalence and seriousness of delirium in critically ill children [5–7], with both hypoactive and hyperactive forms, it is essential for the pediatric critical care community to be able to reliably assess the full spectrum of consciousness in all PICU patients [5–7].

In adults, the Richmond Agitation-Sedation Scale (RASS) provides a single tool that is intuitive, easy to use, and includes both agitation and sedation. The RASS has been shown to be both reliable and valid in critically ill adults with and without mechanical ventilation and sedating medications [8, 9]. It is an attractive option for use as a responsiveness scale in the Pediatric Intensive Care Unit (PICU). However, it has not been validated in children.

We hypothesized that the RASS would be valid in the pediatric population, and could provide clinicians with a way to assess responsiveness in critically ill children.

## Methods

The study took place in a 23-bed urban, academic, tertiary care PICU. Our objective was to include 100 consecutive assessments on designated study days. The RASS had been implemented in this PICU as standard of care approximately 5 years prior and is currently performed every 4 h, on every patient, by nursing staff. For the purpose of this study, a separate (simultaneous) RASS was scored by the research team (*n* = 2). The research team completed an additional sedation assessment, using the previously validated University of Michigan Sedation Scale (UMSS) [10], and the bedside nurse completed a visual analog scale (VAS). Use of the VAS allowed for capture of the bedside nurse’s expert opinion in a quantitative fashion. The UMSS was used to assess criterion validity of the sedation portion of the RASS, as it has been validated in children for procedural sedation. (We chose the UMSS, as other pediatric sedation scores require patient intervention such as testing response to noxious stimuli and cough reflex to suctioning. In contrast, the UMSS is simple and observational and is analogous to the sedation portion of the RASS.) The patient’s sedation regimen was not altered in any way for the purpose of this study.

All patients in the PICU on designated study dates were eligible for inclusion, unless timing was deemed inappropriate by nursing staff due to patient care needs. Exclusion criteria included neuromuscular blockade, quadriplegia, impaired hearing, and impaired visual acuity.

This protocol was approved by the Institutional Review Board of Weill Cornell Medical College, with waiver of consent for this minimal risk observational study.

### Study procedure

Study days were designated by convenience on weekdays when research staff were available. All patients in the PICU on designated study days were included. The nurse and researcher approached the patient’s bedside together. Prior to scoring the RASS, the bedside nurse completed a 10-cm VAS by marking a single line on a scale anchored by the terms “unresponsive” and “combative” for each patient (with the center of the scale intended to represent “alert and calm”). The VAS was completed before the RASS to avoid any influence that the RASS score may have had on the nurse’s assessment of the VAS. The RASS was then scored simultaneously by the bedside nurse and researchers, using the protocol described during the adult validation study [8]. They observed the patient’s level of alertness and agitation for 30 s. If the patient did not meet the criteria for a score of 0–4 (see Table 1), the bedside nurse loudly instructed the patient to “open your eyes,” as the researcher observed. If there was no response, the nurse physically stimulated the patient while saying his/her name. Each evaluator scored the RASS independently based on the guidelines in Table 1 (although the researcher relied upon the nurse’s interaction with the patient in order to generate the RASS score). If the RASS scored by the researcher was −5 to 0, the researcher then evaluated the patient using the UMSS.

**Table 1:** Comparison of Richmond Agitation-Sedation Scale and University of Michigan Sedation Scale

Richmond Agitation-Sedation Scale			University of Michigan Sedation Scale		
Score	Term	Description	Score	Term	Description
4	Combative	Overtly combative or violent; immediate danger to staff			
3	Very agitated	Pulls on or removes tube(s) or catheter(s) or has aggressive behavior toward staff			
2	Agitated	Frequent nonpurposeful movement or patient-ventilator dyssynchrony			
1	Restless	Anxious or apprehensive but movements not aggressive or vigorous			
0	Alert and calm		0	Awake and alert	
−1	Drowsy	Not fully alert, but has sustained (more than 10 s) awakening, with eye contact, to voice	1	Minimally sedated	Tired/sleepy, appropriate response to verbal conversation and/or sound
−2	Light sedation	Briefly (less than 10 s) awakens with eye contact to voice			
−3	Moderate sedation	Any movement (but no eye contact) to voice	2	Moderately sedated	Somnolent/sleeping, easily aroused with light tactile stimulation or a simple verbal command
−4	Deep sedation	No response to voice, but any movement to physical stimulation	3	Deeply sedated	Deep sleep, arousable on with significant physical stimulation
−5	Unarousable	No response to voice or physical stimulation	4	Unarousable	Unarousable

From Sessler et al. [8] and Malviya et al. [10]

### Data collection

For every patient, demographic and clinical data were recorded, including age, gender, Pediatric Index of Mortality (PIM3) [11] score on admission, use and type of respiratory support, and sedative medications administered within 8 h of testing. Data were collected over the course of 4 weeks.

### Statistical analysis

Descriptive statistics were used to describe the patient encounter characteristics, with *N* (%) for categorical variables, and mean +/− SD for continuous variables. To test criterion validity, Spearman’s correlation coefficient was computed to compare scores on the nurse RASS and distance in centimeters of the line drawn on the nurse VAS. Researcher RASS scores and researcher UMSS scores were compared using an equally weighted Cohen’s kappa coefficient if the researcher RASS scores were −5 to 0 (sedation portion). The specific RASS items from 0 to −5, respectively, include “alert and calm,” “drowsy,” “light sedation,” “moderate sedation,” “deep sedation,” and “unarousable.” The UMSS items from 0 to 4, respectively, include “awake and alert,” “minimally sedated,” “moderately sedated,” “deeply sedated,” and “unarousable.” To construct pairs of scores for comparison between the two scales, “drowsy” on the RASS was combined with “light sedation,” and this new group was compared to “minimally sedated” on the UMSS. All other scores matched appropriately for comparison (with the caveat that the UMSS allows for response to touch and not just verbal stimulation). The pairs are shown in Table 1. To test inter-rater agreement for the RASS, the nurse RASS scores and the researcher RASS scores were compared using Cohen’s kappa. To account for possible bias from same-patient encounters, sensitivity analyses were then performed by re-running all tests for only the unique first encounters. Subgroup analyses compared RASS scores in different age groups, between subjects who were sedated at any time in the past 8 h and those who were not, and between mechanically ventilated and spontaneously breathing subjects. Comparisons were made with Wilcoxon rank-sum tests/independent sample *t* tests and Kruskal-Wallis rank-sum tests/ANOVA as appropriate. All statistical tests were two-sided with statistical significance evaluated at the 0.05 alpha level. Analyses were performed with R version 3.2.1 for Windows 64-bit.

## Results

### Patient demographics

One hundred consecutive patient encounters from 50 unique patients were evaluated. Clinical and demographic characteristics of the 100 assessments are summarized in Table 2. The patients ranged in age from 2 months to 21 years, with a median patient age of 2.5 years. Seventy percent of patients were male and 30 % were female. The mean probability of mortality as calculated by the PIM3 score was 3.4 % (median 1.6 %). The 200 RASS scores ranged from −5 to +3. RASS scores were significantly lower in patients who were mechanically ventilated as compared to those who were breathing spontaneously (mean (SD) RASS −1.8 (2.6) and −0.1 (1.3), *p* < 0.001). There was no significant difference in RASS scores between age groups.

**Table 2:** Clinical and demographic characteristics of assessments (*n* = 100)

		*N* (%)
Age	<5 years	65 (65)
	5–10 years	14 (14)
	>10 years	21 (21)
Gender	Male	70 (70)
	Female	30 (30)
Respiratory Support	None	65 (65)
	Invasive mechanical ventilation	27 (27)
	Supplemental oxygen	8 (8)
Sedative medication in prior 8 h	None	57 (57)
	Narcotic alone	14 (14)
	Benzodiazepine alone	6 (6)
	Dexmedetomidine alone	3 (3)
	Narcotic + benzodiazepine	11 (11)
	Narcotic + dexmedetomidine	9 (9)

### Validity

Regarding construct validity, RASS scores were significantly lower in those who had received sedating medications at any time in the past 8 h (mean (SD) RASS −1.3 (2.46) and 0 (1.04), *p* < 0.001).

**Fig. 1:**
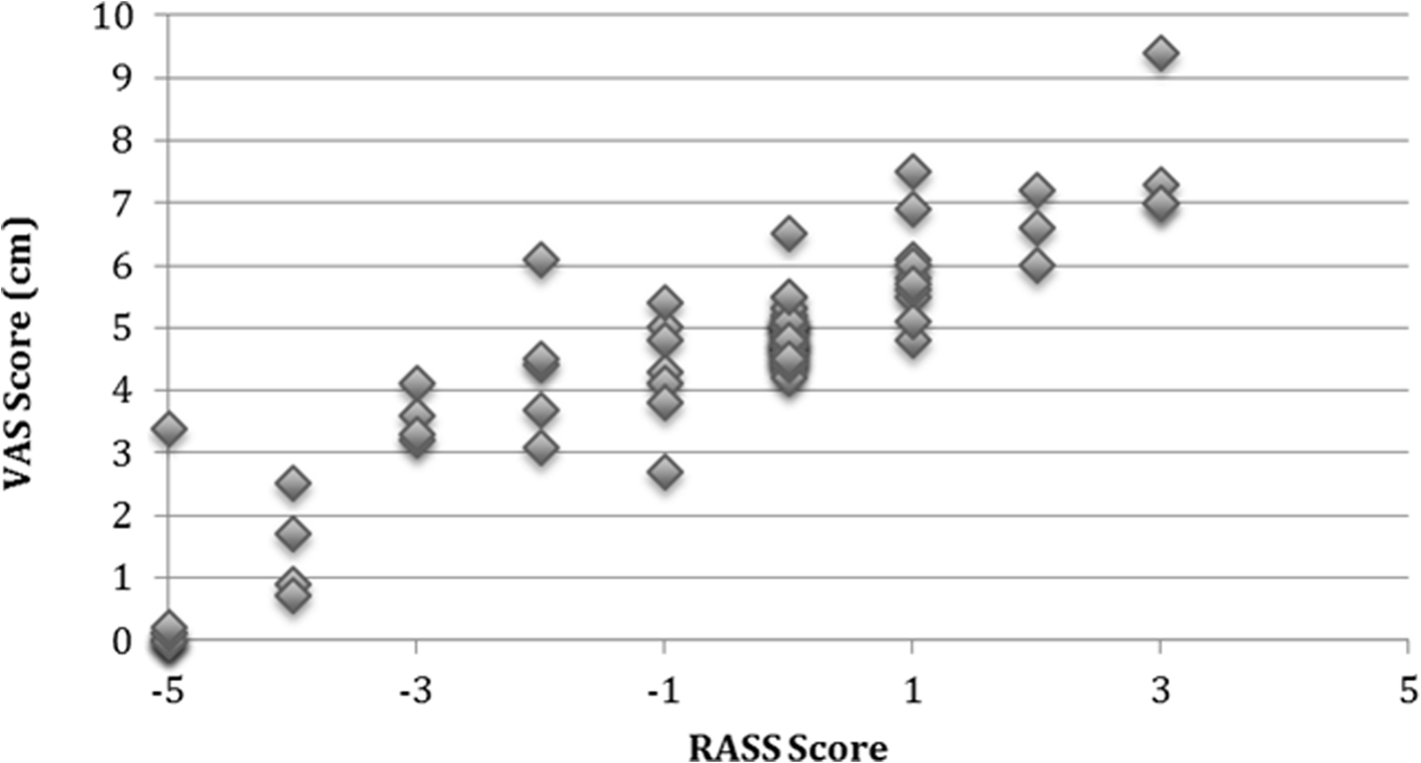
Correlation of nurse VAS and RASS scores (*n* = 97). Legend: nurse-reported RASS scores ranging from −5 to +4 correlated highly with the 10 cm VAS also performed by the patient’s nurse. (*ρ* = 0.810, *p* < 0.0001)

Three encounters did not have nurse sedation-agitation VAS scores recorded, resulting in *n* = 97 for comparison between nurse VAS and nurse RASS. The nurse RASS scores correlated highly with the nurse VAS scores (*ρ* = 0.810, *p* < 0.0001) (Fig. 1). Researcher RASS scores in the sedation range (*n* = 82) showed high agreement with the corresponding researcher UMSS scores (weighted *κ* = 0.902, *p* < 0.0001) (Table 3). In sensitivity analysis, when re-run for only the first unique patient encounters (*n* = 50), the correlation of nurse RASS and nurse VAS scores still remained high (*ρ* = 0.750, *p* < 0.0001), as did the agreement between the researcher RASS and researcher UMSS scores (*κ* = 0.816, *p* < 0.0001).

**Table 3:** Concordance between researcher UMSS and RASS scores (*n* = 82)

	Researcher UMSS				
Researcher RASS	“Unarousable”	“Deeply sedated”	“Moderately sedated“	“Minimally sedated”	“Awake and alert”
“Unarousable”	8	0	0	0	0
“Deep sedation”	0	3	1	0	0
“Moderate sedation”	0	1	4	0	0
“Drowsy/Light sedation”	0	0	5	5	0
“Alert and calm”	0	0	0	3	52

Agreement was high between the nurse and researcher UMSS scores (weighted *κ* = 0.902, *p* < 0.0001)

### Reliability

The mean (SD) RASS scores were −0.56 (1.89) and −0.59 (1.83) for the nurses and researchers, respectively. Among nurse RASS scores, 28 % were in the sedation range, 18 % were in the agitation range, and 54 % were zero. Among researcher RASS scores, 27 % were in the sedation range, 18 % were in the agitation range, and 55 % were zero (Table 4). Agreement was high between the nurse and researcher RASS scores (weighted *κ* = 0.825, *p* < 0.0001). When analyzing only the 50 first encounters, the agreement was excellent (*κ* = 0.936, *p* < 0.0001). When analyzing only infants (*n* = 34), inter-rater agreement remained acceptable (*κ* = 0.873, *p* < 0.001), despite the fact that this population is often considered difficult to assess.

**Table 4:** Agreement between nurse and researcher RASS scores (*n* = 100)

	Nurse RASS								
Researcher RASS	“Unarousable”	“Deep sedation”	“Moderate sedation”	“Light sedation”	“Drowsy”	“Alert and calm”	“Restless”	“Agitated”	“Very agitated”
“Unarousable”	8	0	0	0	0	0	0	0	0
“Deep sedation”	0	3	1	0	0	0	0	0	0
“Moderate sedation”	0	1	2	2	0	0	0	0	0
“Light sedation”	0	0	1	2	0	0	0	0	0
“Drowsy”	0	0	0	0	6	1	0	0	0
“Alert and calm”	0	0	0	1	1	50	2	0	1
“Restless”	0	0	0	0	0	2	9	0	0
“Agitated”	0	0	0	0	0	1	0	3	3
“Very agitated”	0	0	0	0	0	0	0	0	0

Agreement was high between the nurse and researcher RASS scores (weighted *κ* = 0.825, *p* < 0.0001)

## Discussion

The RASS was developed in adult patients, with an emphasis placed on ease of use and clarity [8]. It is an intuitive tool that spans the full spectrum of responsiveness and has been widely adopted internationally for use in critically ill adult patients [9]. Many PICUs currently use the RASS as the standard of care to evaluate responsiveness [12, 13]. However, other PICUs have felt limited, as the RASS had never been formally studied for use in pediatric populations.

RASS scores correlated highly with a VAS scored by the patient’s nurse and showed high concordance with the UMSS scored by the researcher. Current tools used to measure agitation in children have primarily been validated in children who are intubated and/or sedated, and can be difficult to use [1–4]. Because there are currently no simple, observational validated measures of agitation in children, a VAS was used, anchored by “unresponsive” and “combative.” The use of a VAS is an acceptable way to establish validity when there is no gold standard for comparison, and a VAS has been used in similar studies validating responsiveness scales [14]. UMSS is a previously validated tool that assesses sedation only, and was used to measure validity in patients whose RASS fell in the calm or sedated range [10]. These results indicate that the RASS score consistently agrees with the clinical expertise of the patient’s nurse and with a previously validated scale.

Inter-rater agreement between nurse’s RASS and researcher’s RASS was excellent, with all kappa scores >0.8, indicating near-perfect agreement [15]. It must be noted that these were not completely independent scores, as for those children who required verbal or physical stimulation, the researcher relied upon observation of the nurse’s interaction with the patient in order to generate the RASS score. This is similar to the method used to determine nurse inter-rater reliability in critically ill adults [8, 9], but is an important limitation.

With 50 unique subjects included, we have a larger sample size than that used for testing of other widely used pediatric tools [10, 14]. The study population is representative of the distribution of patients in PICUs in the USA, with ~30 % of children on invasive mechanical ventilation and >60 % under age 5 [16]. The assessors in this study included nurses and specially trained research staff, indicating that the tool is reliable across healthcare disciplines. These results indicate that the RASS is a valid tool for use in pediatric patients.

There are a few responsiveness scales that have been tested in pediatric patients [4]. Those that have been developed have important limitations that restrict their usefulness in the PICU. The Comfort Scale and its newer version, Comfort-B, are elegant and comprehensive tools that require a complex system of scoring for multiple indicators and may be cumbersome for frequent use in a busy PICU [1, 2]. The State Behavioral Scale was developed for pediatric patients and tested for reliability and validity; however, the scale was designed for only those patients on mechanical ventilation [3]. A new scale, the Pediatric Sedation-Agitation Scale (P-SAS), is being developed based on the Sedation Agitation Scale for adults. However, this scale has not yet been formally tested for reliability and validity [17]. The UMSS is simple and has been validated in children, but this scale only measures sedation and is therefore not useful in patients who may be agitated [10]. The RASS fills this needed gap.

With validation of the RASS in pediatric patients, use of this tool in PICUs may allow for more accurate assessments of responsiveness and will improve our ability to conduct research on the risk factors and outcomes associated with various levels of sedation and agitation. This will facilitate screening and research in related areas such as delirium, where the RASS has already been used in the validation of delirium scoring systems such as the Cornell Assessment of Pediatric Delirium (CAPD) and the Pediatric Confusion Assessment Method for the ICU (pCAM-ICU) [12, 13].

In practical use, the RASS is not only descriptive but also well-suited for improving sedation titration at the bedside. In our institution, on daily rounds, the medical team determines the “RASS goal,” or targeted level of awareness, for each patient based on their clinical status. The nurse scores the RASS every 4 h (at minimum) and titrates treatment to achieve that goal over the course of the day. For example, a 2-month old baby with acute respiratory failure due to viral bronchiolitis, on moderate ventilator settings without other significant organ dysfunction, would likely have a target RASS of −1. If the child is scored a −2 or −3, the nurse will decrease sedation in order to move the patient toward the RASS goal.

We would argue that the RASS is more accurately called an “awareness scale” rather than an “agitation and sedation scale” because it can be used to assess responsiveness even in patients who are not receiving sedation medications. A child with hypoactive delirium may have a negative RASS score even in the absence of pharmacologic sedation. Our study population included many subjects who were not pharmacologically sedated, as our PICU currently minimizes sedation as per current Society for Critical Care Medicine (SCCM) guidelines [18]. Using the RASS to monitor responsiveness in all critically ill children is clinically useful, because assessing awareness is important for increasing recognition of both hypoactive and hyperactive forms of pediatric delirium.

There are several potential limitations to this study. The RASS measures responsiveness using eye contact and voice as a response to verbal stimulus, so it is not generalizable to patients with severe visual or auditory impairment, and they were necessarily excluded from this study. Additionally, this study was performed in a single institution. In our PICU, our goal is to keep children as awake as possible, even when on mechanical ventilation. By minimizing sedation, we hope to prevent delirium and facilitate early mobilization. As such, these results may not be readily generalizable to other PICUs with more liberal approaches to sedation. Multi-institutional validation would be ideal.

## Conclusions

The RASS is a valid responsiveness scale for use in critically ill children. It is quick and intuitive, making it an excellent tool for use in the PICU.

## Abbreviations

CAPD: Cornell Assessment of Pediatric Delirium
ICU: Intensive care unit
pCAM-ICU: Pediatric Confusion Assessment Method for the ICU
PICU: Pediatric intensive care unit
PIM3: Pediatric Index of Mortality 3
RASS: Richmond Agitation-Sedation Scale
SCCM: Society for Critical Care Medicine
UMSS: University of Michigan Sedation Scale
VAS: Visual analog scale
